# Supplementary material for: Using a theory-informed approach to explore patient and staff perspectives on factors that influence clinical trial recruitment for patients with cirrhosis and small oesophageal varices
Source: PLoS One. 2022 Feb 3;17(2):e0263288. doi: 10.1371/journal.pone.0263288 (PMC8812916; doi:10.1371/journal.pone.0263288)
Supplement: S1 File — (DOCX) [file pone.0263288.s001.docx]

**Interview recruitment for patients who agree or decline to take part in the BOPPP Trial**

Once a patient has agreed or declined to take part in the BOPPP Trial, the research team will propose the qualitative interview and a Participant Information Sheet (PIS) will be provided.

Participant identification initiated at site level by the research team during the screening process for BOPPP Trial participation.

Once successful contact has been established between patient and researcher, the site and the researcher will delete the email. The patient’s contact details will be stored in a password protected document on a password protected PC at KCL.

A copy of the ICF will be forwarded to the participant. The participant’s unique study identifier and date of consent will be forwarded to site for accrual (as required).

The patient declines to take part.

The researcher will document the patient’s decision to decline on the appropriate log.

The patient declines to be contacted by the researcher.

The research team will document the patient’s decision to decline on the appropriate log.

The original ICF will be stored with the qualitative researchers at King’s College London.

Consent will be obtained by the researcher using the Informed Consent Form (ICF) – either face-to-face or by telephone. If by telephone, the researcher will initial each consent item on behalf of the patient. The conversation will be audio-recorded and later observed by a witness and countersigned to confirm that informed consent was taken.

The researcher will follow up with the patient e.g. telephone or email, to obtain and document the patient’s decision to agree/decline to take part in the interview.

The research team will forward patient contact details (by phone or email using a secure “nhs.net” to “nhs.net” email account) to the researcher.

The research team will obtain verbal consent from the patient to forward their contact details to the qualitative researcher at KCL. The researcher’s contact details will also be provided for the patient to contact the researcher directly.

**Interview recruitment for staff who recruit patients to the BOPPP Trial**

Participant identification initiated by the BOPPP Trial Manager/Site PIs. All research staff responsible for recruiting patients to the BOPPP Trial are eligible.

Once successful contact has been established, the site and researcher will delete the email. The participant’s contact details will be stored in a password protected document on a password protected PC at KCL.

The potential participant declines to take part.

The qualitative researcher will document the decision to decline on the appropriate log.

The qualitative researcher will make contact with the identified potential participants (by phone or email using a secure “nhs.net” to “nhs.net” email account) to propose the qualitative interview, to provide a Participant Information Sheet (PIS) and to answer questions. The researcher will obtain and document the decision to agree/decline to take part in the interview.

A copy of the ICF will be forwarded to the participant. The participant’s unique study identifier and date of consent will be forwarded to site for accrual.

Consent will be obtained by the researcher using the Informed Consent Form (ICF) – either face-to-face or by telephone. If by telephone, the researcher will initial each consent item on behalf of the participant. The conversation will be audio-recorded and later observed by a witness and countersigned to confirm that informed consent was taken.

The original ICF will be stored with the qualitative researchers at King’s College London.
