# Supplementary material for: Using a theory-informed approach to explore patient and staff perspectives on factors that influence clinical trial recruitment for patients with cirrhosis and small oesophageal varices
Source: PLoS One. 2022 Feb 3;17(2):e0263288. doi: 10.1371/journal.pone.0263288 (PMC8812916; doi:10.1371/journal.pone.0263288)
Supplement: S2 File — (DOCX) [file pone.0263288.s002.docx]

Patient Interview Schedule: BOPPP Trial

*The aim of this interview is to explore patient perceptions regarding recruitment procedures and perceived benefits and disadvantages of taking part in a research trial. Participants will be encouraged to discuss their opinions, experiences and perceptions in an open way to ensure any issues of importance to the BOPPP trial are not excluded. The information gathered will be used to improve the recruitment process, information about the trial, and support offered to prospective participants and patients who go on to participate in the BOPPP trial.*

*The interview schedule contains key questions and themes to be explored. Further questioning will fully explore participants’ contributions in order to understand how and why views are held.*

Participant information sheet provided in advance. Participants given the opportunity to ask questions, and sign consent form.

1. Introduction to interview and ground rules

*Aim: to introduce the research and set the context for the interview*

- **Introduce interviewer**
- **Introduce the interview study**

The purpose of the interview study is to find out about your views and experiences on the BOPPP research trial regardless of whether you agreed or declined to take part. This provides us with information on factors that influence the decision-making process about whether or not to take part, and will be used to improve the research design, information, and support provided to prospective participants in research trials of this sort.

- **Details about participation**

Voluntary nature of participation – both overall and in relation to any specific questions and discussions

- - Length of interview – around 1 hour. Will finish on time – clock to keep to time
  - Recording of interview – stop for loud noises
  - Confidentiality and how findings will be reported - anonymity, transcribing
  - Emphasise there is no need to share personal experiences unless people want to
  - Explain that there are no right or wrong answers – interested in a range of views,

opinions and experiences

- **Basic ground rules**
  - Mobile phones off (or on silent/vibrate)
- Any questions?

1. Background

*Aim: to introduce context of the BOPPP trial and recruitment procedure*

- To start, we will provide a brief overview of the BOPPP trial and the research recruitment procedure.

1. Introduction for participant – **Start recording session**

- Our overall aim is to understand your experience of being invited to take part in a research trial. We are interested in why or why not you decided to participate in the BOPPP trial. Before we start, we would like to find out about your experience of living with cirrhosis. Can you tell me about that?

1. Understanding barriers and facilitators to trial recruitment

*Aims: 1) To explore participants experiences of the recruitment process.*

*2) To explore why participants choose to decline or take part in the BOPPP trial.*

3a. **What helps recruitment?** Facilitating factors

*Link: We’ve been hearing about the BOPPP trial, I’d like to start by asking…*

- **about your experience of the research recruitment process and being invited to take part in the BOPPP trial What do you think about taking part in a research trial?**
- **What are the perceived benefits of taking part?**
- **What influenced your decision to take part?**
- **What are your views on the recruitment screening, consent, baseline visit and one-week review?**

Prompts:

- - acceptability of trial/recruitment procedures/randomisation
  - benefits and risks of trial intervention and participation
  - Please tell us about your experience of the BOPPP treatment intervention
  - how study was presented and study information

*Probe:*

- **What can you tell us about that?**
- **Are there any other factors that help?**
- Bring it back on topic… **Can I ask a question related to that, that I asked at the beginning – what helps you to recruit patients to the BOPPP trial?**
- Uncertainty of participant in answering question… **Which way is most important to you?**

3b. **What prevents or hinders recruitment?** Barriers (25 mins)

- **What are the perceived disadvantages of taking part in the trial?**
- **What are your concerns about taking part?**
- **What influenced your decision not to take part?**

Prompts:

- - acceptability of trial/recruitment procedures/randomisation
  - benefits and risks of trial intervention and participation
  - how study was presented and study information

*Probe:*

- **What can you tell us about that?**
- **Are there any other factors that hinder?**
- Bring it back on topic… **Can I ask a question related to that, that I asked earlier – what hinders you/your team from recruiting trial participants?**
- Uncertainty of participant in answering question… **Which way is most important to you?**

3c. Recruitment solutions (25 mins)

- **How could this research study and others of this type be improved?**
- **Are there any strategies or potential solutions that might support your decision to take part in other future research of this type?**
- **This interview was designed to help us understand why or why not you decided to take part in the BOPPP trial. Are there any other important points that you would like to discuss before we close the discussion?**

Bring discussion to a close, thank respondents and reiterate confidential nature of group. Any further questions about us or the research?

Explain what happens next – involvement in RCT and when they might next hear from BOPPP.

Staff Interview Schedule: BOPPP Trial

*The aim of this interview is to explore staff perceptions regarding recruitment procedures and perceived benefits and disadvantages of recruiting patients to a clinical trial, as well as gathering views on the use of beta-blocker treatment for patients with small oesophageal varices in routine practice. Participants will be encouraged to discuss their opinions, experiences and perceptions in an open way to ensure any issues of importance to the BOPPP trial are not excluded. The information gathered will be used to improve: the research design and structure of the recruitment process, information about the trial, and support offered to prospective participants and patients who go on to participate in research trials of this sort.*

*This interview schedule contains key questions and themes to be explored. Further questioning may arise following participant responses that are not detailed within this guidance document. This is permitted and will be necessary to fully explore participants’ contributions in order to understand how and why views are held. Paraphrasing of any of the suggested wording is allowed to accommodate personal communication preferences.*

Participant information sheet provided in advance. Participants given the opportunity to ask questions, and sign consent form.

1. Introduction to interview and ground rules

*Aim: to introduce the research and set the context for the interview*

- **Introduce interviewer**
- **Introduce the interview study**

The purpose of the interview study is to find out about your views and experiences of recruiting participants to the BOPPP trial. This provides us with information on factors that influence the decision-making process about whether or not to take part and will be used to improve the research design, information, and support provided to prospective participants in research trials of this sort. We are also interested in finding out about your views and experiences of implementing the BOPPP trial treatment strategy as a part of routine clinical care. This provides us with information on factors that facilitate or hinder implementation and on possible solutions to overcome any identified barriers.

- **Details about participation**

Voluntary nature of participation – both overall and in relation to any specific questions and discussions

- - Length of interview – around 30-60 mins. Will finish on time – clock to keep to time
  - Recording of interview – stop for loud noises
  - Confidentiality and how findings will be reported - anonymity, transcribing
  - Emphasise there is no need to share personal experiences unless people want to
  - Explain that there are no right or wrong answers – interested in a range of views, opinions and experiences
- **Basic ground rules**
  - Mobile phones off (or on silent/vibrate)
- Any questions?

1. Background

*Aim: to introduce context of the BOPPP trial and recruitment procedure*

- To start, we will provide a brief overview of the BOPPP trial and the context of beta-blocker use as defined by this study. We will also provide a brief overview of the recruitment procedure for the BOPPP trial.
- Pre-screening of clinic and endoscopy records undertaken by local clinical teams to identify suitable patients
- Treating clinical team introduces trial to patient and obtains verbal consent for patient to be contacted by research team
- Participant information sheet (PIS) and written informed consent form supplied to patients who agree to be contacted (by research team) and a follow-up screening appointment arranged
- Patients will have the opportunity to read the PIS and ask questions regarding their participation/the trial at the screening visit
- Additional time will be offered for those who require it before making a decision whether or not to take part.

1. Introduction for participant – **Start recording session**

*Link: Our overall aim is to understand the facilitators and barriers of trial recruitment but we would like to start by asking...*

- **What do you think about using beta-blockers with this group of patients? Are there any benefits? Or challenges?**
- **What helps you (and your colleagues) to provide beta-blocker treatment as a part of routine clinical care?**
- **What prevents or hinders success in implementing the beta-blocker treatment strategy as a part of routine clinical care?**

Prompts based on the Theoretical Domains Interview (Michie 2005) will support brief discussion around barriers and facilitators based on the listed domains. The list will not be followed in a predetermined order.

- - Knowledge: Are you familiar with this drug treatment? And in this context?
  - Skills: Do you know how to monitor/titrate the treatment?
  - Social/professional role and identity: Might this treatment and/or research trial challenge your professional role?
  - Beliefs about capabilities: How well equipped or confident are you that you can provide this treatment?
  - Beliefs about consequences: Do you believe that implementing this treatment is a good thing?
  - Motivation and goals: Are there other things that you would like to do or achieve that might interfere with this treatment?
  - Memory, attention and decision-making processes: Do you think you will have to pay more attention to provide this treatment and will you remember to do it?
  - Environmental context and resources: To what extent do physical or resource factors facilitate or hinder you from implementing the treatment?
  - Social influences: To what extent do social influences facilitate or hinder you from implementing the treatment (peers, managers, other professional groups, patients, carers)?
  - Emotional regulation: To what extent do emotional factors facilitate or hinder?

1. Understanding barriers and facilitators to trial recruitment

*Aims:*

*1) To explore what participants think helps or hinders recruitment.*

*2) To explore what participants identify as potential solutions to recruitment barriers.*

3a. What helps recruitment? Facilitating factors

*Link: We’ve been hearing about the BOPPP trial,*

- **We are also keen to hear about your views on the BOPPP trial. Do you think the study is important? How might the findings add to existing knowledge?**
- **What do you think about recruiting participants into a research trial?**
- **What examples do you have of successfully recruiting patients to the trial?**
- **What is it that enables you to successfully recruit to the BOPPP trial?**
- **What are the influences at patient-level? Or research-site level? Anything else?**
- **Can you tell us about how you might identify suitable participants for the trial?**
- **Can you tell us about your thoughts on randomisation? For example, might there**

**be concerns about allocating a patient with a small varix to the control group?**

Prompts:

- - acceptability of trial/recruitment procedures/randomisation
  - benefits and risks of trial intervention and participation
  - Please tell us about your experience of the BOPPP treatment intervention

*Probe:*

- **What can you tell us about that?**
- **Are there any other factors that help?**
- Bring it back on topic… **Can I ask a question related to that, that I asked at the beginning – what helps you to recruit patients to the BOPPP trial?**
- Uncertainty of participant in answering question… **Which way is most important to you?**

3b. What prevents or hinders recruitment? Barriers

- **What prevents or hinders success in recruiting patients to the BOPPP trial?**
- **What would further support you to recruit participants?**
- **What are the influences at patient-level? Or research-site level? Anything else?**
- **How likely is it that you might want to withdraw a recruited patient from the trial? Can you give us an example of when you might consider withdrawing a patient from the trial?**

Prompts:

- - acceptability of trial/recruitment procedures/randomisation
  - benefits and risks of trial intervention and participation
  - What resources would better equip/support you to recruit patients to the trial?
  - Please tell us about your experience of the BOPPP treatment intervention and any perceived challenges of this treatment strategy in routine practice

*Probe:*

- **What can you tell us about that?**
- **Are there any other factors that hinder?**
- Bring it back on topic… **Can I ask a question related to that, that I asked earlier – what hinders you/your team from recruiting trial participants?**
- Uncertainty of participant in answering question… **Which way is most important to you?**

3c. Recruitment solutions

- **What solutions would you recommend to address these identified barriers?**
- **Are there any strategies and potential solutions to optimise recruitment and retention?**

Prompts:

- Behavioural regulation: What preparatory steps are needed for you/your colleagues to optimise recruitment? What information is needed to support recruitment/improve uptake?
- Nature of the behaviour: Who needs to do what differently when, where, how, how often and with whom?
- **This interview was designed to help us understand the extent to which the BOPPP trial recruitment procedures support successful recruitment and retention. Are there any other important points that you would like to discuss before we close the discussion?**

Bring discussion to a close, thank respondents and reiterate confidential nature of group.

Any further questions about us or the research?

Explain what happens next – involvement in focus groups/RCT and when they might next hear from BOPPP.
