# Supplementary material for: Using a theory-informed approach to explore patient and staff perspectives on factors that influence clinical trial recruitment for patients with cirrhosis and small oesophageal varices
Source: PLoS One. 2022 Feb 3;17(2):e0263288. doi: 10.1371/journal.pone.0263288 (PMC8812916; doi:10.1371/journal.pone.0263288)
Supplement: S3 File — (DOCX) [file pone.0263288.s003.docx]

**Prioritising BOPPP and optimising trial recruitment**

Coding framework

**Theme 1: Patient risks and benefits**

- 1. **Acceptability of the trial**
     1. *Three-year commitment*
        1. *Extra appointments and blood tests*
           1. *Fitting appointments into existing clinical appointments/work commitments*
     2. *Finding out about the trial at the right time*
        1. *Too soon after diagnosis*

*1.1.2.2 Timing at endoscopy does not suit everyone*

- - 1. *Existing relationships and confidence in the team*
    2. *Understanding the information*

*1.1.4.1 Face-to-Face interaction and simple language*

*1.1.4.2 Time to understand the information*

- 1. **An opportunity to help myself and others**
     1. *Invested in my own health*
        1. *A chance to be on treatment*
        2. *Placebo effect as a positive*
        3. *Regular clinic appointments and more timely treatment*
           1. *Health is being monitored*
     2. *Reassurance to family and staff as ‘seen to be helping self’*
     3. *Giving back to the clinical team*
     4. *Helping others and the greater good*
        1. *Involvement in future treatment options*
     5. *It’s part of my routine now*
  2. **Uncertainty and too much of a commitment**
     1. *All effort is on managing existing health needs*
     2. *Uncertainty about treatment*
        1. *Side effects*
        2. *Another tablet*
        3. *Risk of placebo when treatment might help*

**Theme 2: Staff attitudes, knowledge and capacity**

- 1. **Staff attitudes**
     1. *Perceived value of BOPPP*
        1. *BOPPP adds to care*
        2. *BOPPP will provide evidence for treatment*
        3. *BOPPP is an ‘easy’ intervention*
        4. *A phase 4 trial*
        5. *Clinical equipoise*
           1. *Views on effectiveness of intervention*
        6. *Views on research*
           1. *All patients should have access to trials*
           2. *Too many research studies*
     2. *BOPPP means asking more of patients*
        1. *Extra appointments and blood tests*
           1. *Time off work*
           2. *Additional travel*
           3. *Caring responsibilities*
        2. *Concern about providing extra clinical input in stable disease*
           1. *Challenges of prescribing NSBBs*

*1.1.2.2.2 Impact of side effects on quality of life*

- - 1. *Predicting patient suitability*
       1. *Patients who are active in their own care*
       2. *Research is a hassle*
          1. *Engaging the ‘other’ demographic*

*Lifestyle*

*Those of no further address*

*Older people*

*2.1.3.3 Conflicts with clinical responsibilities*

- 1. **Staff knowledge, experience, and skills**

*2.2.1 Perceptions of knowledge*

*2.2.1.1 Eligibility criteria*

*2.2.1.2 Hepatology and proposed intervention*

*2.2.1.2.1 Liver disease is not a speciality*

*2.2.1.3 Research processes*

- - - - 1. *New to research*
    1. *Confidence and ability to screen and identify patients*
    2. *Effective communication with eligible patients*
       1. *Able to answer questions about BOPPP*

*2.2.3.2 Able to provide accurate and accessible information*

- - - - 1. *Language barriers – no interpreters*

*2.2.3.2.2 Involve family*

*2.2.3.3 Providing the right information at the right time*

*2.2.3.3.1 At which point the PIS is provided*

*2.2.3.3.2 Working around priorities of patients (i.e. work/family commitments)*

*2.2.3.4 Opportunity to meet face-to-face Vs telephone*

*2.2.3.4.1 Impact of ‘cold calling’*

- 1. **Staff capacity**

*2.3.1 BOPPP adds to the workload*

*2.3.1.1 Consider the amount of work required for each BOPPP participant*

*2.3.1.2 Consider how BOPPP fits in with existing workloads*

*2.3.1.2.1 Prioritising BOPPP when working on between one and fifteen studies*

**Theme 3: Team-based approach**

**3.1 Shared views and goals**

*3.1.1 Team-level understanding of BOPPP*

*3.1.2 Prioritising BOPPP*

**3.2 Team coordination of BOPPP tasks**

*3.2.1 Deliberate organisation of BOPPP tasks*

*3.2.1.1 Pre-plan BOPPP time*

*3.2.1.2 Identify which team members are responsible for which tasks*

*3.2.1.2.1 Familiarity of staff supports recruitment*

*3.2.1.2.1.1 Patients may want to speak to their doctor before deciding*

*3.2.1.2.1.2 Research staff are less familiar than clinical staff*

*3.2.2 Team time to reflect on the recruitment effort*

**3.3 Effective team communication**

*3.3.1 Shared communication across teams*

*3.3.1.1 The degree to which research and clinical teams co-exist*

*3.3.1.1.1 Research staff are not always embedded in clinical team*

*3.3.1.1.2 Team relationships with pharmacy, endoscopy*

**3.4 Team leadership**

*3.4.1 PI involvement and support*

**Theme 4: Organisational context**

**4.1 Organisational culture and leadership**

*4.1.1 Trial is well promoted and supported within organisation*

*4.1.1.1 Trial promoted to staff in local teaching sessions*

*4.1.2 Degree to which other aspects of care delivery conflict or align with BOPPP*

*4.1.2.1 Amount of time available for endoscopies*

**4.2 Organisational resources**

*4.2.1 Time*

*4.2.1.1 Limits on PI/clinician time*

*4.2.1.1.1 Own time or additional clinic time for BOPPP visits*

*4.2.1.2 Screening is time-intensive*

*4.2.1.3 Time conflicts between research and clinical responsibilities*

*4.2.1.3.1 BOPPP is not grounded in existing clinical practice*

*4.2.2 Financial resources*

*4.2.2.1 Travel costs*

*4.2.2.2 Translator costs*

*4.2.3 Space*

*4.2.3.1 Rooms available for BOPPP visits*

*4.2.3.1.1 Difficulty booking rooms around clinic times*

*4.2.4 Workforce*

*4.2.4.1 Availability and assignment of staff for BOPPP tasks*

**4.3 Organisational workflows**

*4.3.1 Variability in local eligibility screening processes*

*4.3.1.1 Endoscopy is the golden time*

*4.3.1.2 Joint screening with other studies sometimes helpful*

*4.3.2 Fewer than expected eligible patients*

*4.3.2.1 Pre-existing conditions*

*4.3.2.2 Already prescribed beta-blockers*

*4.3.2.3 Contra-indications of beta-blockers*

*4.3.3 Scheduling and timeframes*

*4.3.3.1 Patients outside the endoscopy window*

*4.3.3.2 Difficult to line up the ducks*

*4.3.3.3 Randomisation process takes time*

**Theme 5: Trial collective**

**5.1 Feeling part of the BOPPP team across sites**

*5.1.1 Links with Chief investigator and/or Lead site*

*5.1.2 Communication with the central team*
