# Supplementary material for: Using a theory-informed approach to explore patient and staff perspectives on factors that influence clinical trial recruitment for patients with cirrhosis and small oesophageal varices
Source: PLoS One. 2022 Feb 3;17(2):e0263288. doi: 10.1371/journal.pone.0263288 (PMC8812916; doi:10.1371/journal.pone.0263288)
Supplement: Attachment — Submitted filename: Response to Reviewers.docx [file pone.0263288.s004.docx]

Dear Editor	16/11/2021

**Re: Using a theory-informed approach to explore patient and staff perspectives on factors that influence clinical trial recruitment for patients with cirrhosis and small oesophageal varices**

Thank you very much for reviewing this manuscript. Please find responses to each point raised by the academic editor and reviewer(s) below. All changes to the manuscript are highlighted with track changes.

1. Please ensure that your manuscript meets PLOS ONE's style requirements, including those for file naming.

*We have checked PLOS ONE’s style requirements and have changed the supplementary information file names to meet the requirements.*

2. When reporting the results of qualitative research, we suggest consulting the COREQ guidelines. In this case, please consider including more information on the number of interviewers, their training and characteristics; and please provide the interview guide used.

*We have used the COREQ guidelines to report the study – and re-attach the checklist. We have included information on the number of interviewers and the two interview schedules are included in supplementary information 2. We have highlighted where this information is available in the text using* ***bold.*** *We have extended the information to include the training and characteristics of the interviewer (page 7).*

3. Please provide additional details regarding participant consent. In the ethics statement in the Methods and online submission information, please ensure that you have specified whether consent was written or verbal/oral. If consent was verbal/oral, please specify: 1) whether the ethics committee approved the verbal/oral consent procedure, 2) why written consent could not be obtained, and 3) how verbal/oral consent was recorded.

*We have included additional information regarding participant consent – and clarified that patients were offered face-to-face or telephone interviews. We have confirmed that* *ethical approval was obtained to allow for written and verbal consent and have outlined the procedures (page 7).*

4. Thank you for stating the following in the Acknowledgments Section of your manuscript:

“We would like to thank all patients and staff who participated in this study and who generously gave their time and honest thoughts. We are very grateful to the local BOPPP research teams that helped to recruit participants and supported the qualitative study. This article presents independent research funded by NIHR Health Technology Assessment (17/32/04). The views expressed are those of the author(s) and not necessarily those of the NIHR or the Department of Health and Social Care.”

*We have removed all funding information from the manuscript and ensured the funding information in the Funding Statement section of the online submission form is correct.*

“This article presents independent research funded by NIHR Health Technology Assessment (17/32/04). The views expressed are those of the author(s) and not necessarily those of the NIHR or the Department of Health and Social Care.”

5. We note that you have stated that you will provide repository information for your data at acceptance. Should your manuscript be accepted for publication, we will hold it until you provide the relevant accession numbers or DOIs necessary to access your data. If you wish to make changes to your Data Availability statement, please describe these changes in your cover letter and we will update your Data Availability statement to reflect the information you provide.

*Thank you for bringing this to our attention. We would like the data availability statement to read:*

*The data that supports the findings of this study are available from the corresponding author [CL] upon reasonable request.*

6. Your ethics statement should only appear in the Methods section of your manuscript. If your ethics statement is written in any section besides the Methods, please delete it from any other section.

*We have checked the manuscript and the ethics information is only included in the methods section.*

7. Please upload a copy of Figure 1, to which you refer in your text on page 7. If the figure is no longer to be included as part of the submission please remove all reference to it within the text.

*Figure 1 has now been uploaded as supplementary information file 1.*

8. Please include captions for your Supporting Information files at the end of your manuscript, and update any in-text citations to match accordingly. Please review your reference list to ensure that it is complete and correct. Any changes to the reference list should be mentioned in the rebuttal letter that accompanies your revised manuscript. If you need to cite a retracted article, indicate the article’s retracted status in the References list and also include a citation and full reference for the retraction notice.

*We have included captions for the supplementary files (S1-S3) at the end of the manuscript. We have reviewed the reference list to ensure it is complete and correct. We have not made any changes to the reference list.*

9. More points regarding the inclusion and exclusion criteria of the trial will enhance the study.

*This information has now been included (page 3).*

10. It can be beneficial in mentioning what kind of questions were asked in interview along with mentioning the results of the participants.

*We have outlined patient questions in the text and have highlighted where this information is available in the text using* ***bold.*** *We have added information about the kind of questions asked in staff interviews (p.7). The interview schedules are included in Supplementary information file 2.*

11. Mentioning more personal comments from the patients or staff or team members regarding individual subthemes can enhance the study further.

*Because of space limitations, the sub-theme findings have been reported within the overarching theme. For example, one sub-theme of patient risks and benefits is acceptability of the trial. This is illustrated by data that explains how a three-year commitment influenced patients’ choice of whether to enrol in the trial (p.17). Another example is the perceived value of BOPPP and views to research as a sub-theme of staff attitude (page 20-21).*

We hope that these responses and changes make the paper suitable for publication in PLOS ONE.

Yours faithfully,

Clair Le Boutillier
