# Supplementary material for: Using a theory-informed approach to explore patient and staff perspectives on factors that influence clinical trial recruitment for patients with cirrhosis and small oesophageal varices
Source: PLoS One. 2022 Feb 3;17(2):e0263288. doi: 10.1371/journal.pone.0263288 (PMC8812916; doi:10.1371/journal.pone.0263288)
Supplement: Attachment — Submitted filename: Response to Reviewers_1.docx [file pone.0263288.s005.docx]

Dear Editor	10/01/2022

**Re: Using a theory-informed approach to explore patient and staff perspectives on factors that influence clinical trial recruitment for patients with cirrhosis and small oesophageal varices**

Thank you very much for reviewing this manuscript. Please find responses to each point raised by the reviewer(s) below. All changes to the manuscript are highlighted with track changes.

1. Has the statistical analysis been performed appropriately and rigorously?

*We have conducted a qualitative study and have therefore not performed any statistical analysis.*

2. The authors should address issue of cultural factors and recruitment of ethnically diverse participants. This is critical issue; cultural and ethnic barriers might need more considerations. If data is not available on diverse participants, authors can use articles from literature to discuss more about this issue in the discussion section.

*We have included this information in the Limitations section of the Discussion – and below for your reference (page 33).*

*It is also important to note, that despite focused efforts to purposively recruit patient participants from minority ethnic backgrounds, experiences of those from these groups are underrepresented. This recruitment difficulty is echoed in literature where it is reported that those from ethnic minorities are less likely to participate in research (Smart & Harrison, 2016). While we were unable to specifically explore ethnic variations and barriers to trial recruitment, we found that researcher bias and judgement of patient suitability dictated those who are prioritised for recruitment. Another possible reason for non-participation is the risk that asking patients to take part in an additional qualitative study may inadvertently alienate them from their enrolment/retention in the clinical trial (Liu et al, 2011). Staff also reported that the quality of relationships with patients is sometimes impacted by language barriers. Future strategies such as availability of interpreters, linguistic and ethnic matching, and an awareness of cultural practices and norms provide possible options for addressing these barriers (Masood et al, 2019).*

3. Similarly, authors should also discuss the issue of relationship between recruiter and study participants, which could be impacted by lingual, cultural and ethnic barriers in more culturally and ethnically diversified trials.

*Thank you for this important point. We have added the need to the impact of the recruiter-study participant relationship (text above) in the discussion (page 33).*

4. The word “Registrar” in Table 3 may need more clarity for international readers.

*Thank you for bringing this to our attention. A speciality registrar is a doctor who is working as part of a training programme in the UK. We have added this definition in brackets for clarification (page 11).*

5. Education levels in Table 2 are categorized as “Full-time education”, “Further education”, “Higher education”. “Post-graduate education”. These names of classification read vague, examples within bracket in fact provide more helpful information. Renaming each of levels of literacy may help bringing more clarity.

*We have added examples within brackets next to each category label (Page 10).*

6. When reporting the results of qualitative research, we suggest consulting the COREQ guidelines. In this case, please consider including more information on the number of interviewers, their training and characteristics; and please provide the interview guide used.

*We have used the COREQ guidelines to report the study – and re-attach the checklist. We have included information on the number of interviewers and the two interview schedules are included in supplementary information 2. We have highlighted where this information is available in the text using* ***bold.*** *We have extended the information to include the training and characteristics of the interviewer (page 8).*

We hope that these responses and changes make the paper suitable for publication in PLOS ONE.

Yours faithfully,

Clair Le Boutillier
